# Premature ovarian insufficiency and early menopause: understanding the aetiology, protecting long-term health and what to expect from future research

**DOI:** 10.1530/RAF-26-0152

**Published:** 2026-10-15

**Authors:** Laura Cervini, Melanie C Davies

**Affiliations:** ^1^ University College London Elizabeth Garrett Anderson Institute for Women’s Health, University College Hospital, London, United Kingdom

**Keywords:** premature ovarian insufficiency, early menopause, hormone replacement therapy, bone health, fertility preservation

## Abstract

**Abstract:**

Premature ovarian insufficiency (POI) and early menopause, defined as ovarian failure before the age of 40 years and menopause between the ages of 40 and 45 years, respectively, affect a considerably higher proportion of people than previously estimated, with a worldwide prevalence of approximately 3.7 and 12%, respectively. Their aetiology is multifactorial, encompassing genetic causes, such as Turner’s syndrome and *FMR1* premutation carrier status; autoimmune conditions; iatrogenic factors, including chemotherapy, radiotherapy and pelvic surgery; and environmental and lifestyle influences, although a substantial proportion of cases remain idiopathic. POI and early menopause are associated with elevated risks of cardiovascular disease, osteoporosis, cognitive decline and dementia, as well as considerable psychological morbidity, including depression, anxiety and fertility-related distress. Many women also experience genitourinary syndrome of menopause (GSM), comprising vulvovaginal and lower urinary tract symptoms arising from hypo-oestrogenism, such as dryness, dyspareunia and recurrent urinary tract infections, alongside reduced libido, with important consequences for quality of life and sexual health. Prompt diagnosis of POI, requiring clinical suspicion alongside biochemical confirmation with elevated follicle-stimulating hormone (FSH) measurement, is important, as diagnostic delays can be associated with a greater bone mineral density loss. Hormonal therapy is the mainstay of management and should be continued until the average age of natural menopause. A comprehensive management approach should include lifestyle interventions and psychosocial support. Significant research gaps remain, including the identification of optimal hormonal regimens or validated biomarkers for early detection. Addressing these and other priorities is essential to improve long-term outcomes and quality of life for people affected by POI and early menopause.

**Lay summary:**

Premature ovarian insufficiency (POI) occurs when the ovaries stop working normally before the age of 40 years. Early menopause means menopause happens between the ages of 40 and 45 years. These conditions are more common than previously thought. In addition to causing menopausal symptoms, such as hot flushes, mood changes and sleep disturbances, these conditions can have serious long-term effects on heart health, bone strength, memory and mental well-being. They can also have a profound emotional impact, particularly on patients who had hoped to have children. Hormone therapy is the main treatment for these conditions and is usually continued until the average age of natural menopause. Care should also include lifestyle support and psychological support. More research is still needed to identify the best hormone therapy options and better ways to detect these conditions early. Addressing these gaps could improve long-term health and quality of life for people with POI or early menopause.

## Introduction

Premature ovarian insufficiency (POI) and early menopause are characterised by the cessation of normal ovarian function before the age of natural menopause. POI is defined as the emergence of ovarian insufficiency prior to the age of 40 years, which is two standard deviations below the mean age of natural menopause, whereas early menopause is defined as menopause occurring between the ages of 40 and 45 years (Panay *et al.* 2020, 2024, Mishra *et al.* 2024).

The progression from ovarian insufficiency to definitive menopause may require months to years. Given that intermittent ovarian activity can occur in POI, accounting for spontaneous pregnancy rates of around 5% (Fraison *et al.* 2019), there has been a shift away from the term ‘premature ovarian failure’ (POF), also due to its negative connotations (Welt 2008). The term ‘primary ovarian insufficiency’ was also employed previously but is not recommended owing to its potential for confusion with primary amenorrhoea.

Early menopause is often overlooked and understudied, and unlike POI, there are no formal guidelines. Mishra proposed viewing this condition as part of a continuous spectrum between POI and natural menopause, rather than a distinct entity (Mishra *et al.* 2024).

A meta-analysis performed by Golezar *et al.* estimated the worldwide prevalence of POI at 3.7% and of early menopause at 12% (Golezar *et al.* 2019). These figures are higher than previous estimates, which typically cited a 1% prevalence for POI (Coulam *et al.* 1986) and a 5% prevalence for early menopause, possibly due to a larger sample size, wider geographic distribution and inclusion of postmenopausal women only. Interestingly, POI prevalence is notably higher in medium and low Human Development Index (HDI) countries. This is thought to be due to biological, socio-economic and lifestyle factors (Golezar *et al.* 2019). In fact, a new pooled analysis of 716,648 women across 44 low- and middle-income countries using Demographic and Health Survey data reported a pooled prevalence of premature or early menopause of 7.1% (95% CI: 7.0–7.2%), with substantial country-level variation (from 2.3% in Jordan to 12% in Ethiopia) and a consistently higher burden in rural areas (Islam *et al.* 2026). This more recent estimate reinforces that these conditions are unevenly distributed globally and adds to the evidence that POI and early menopause historically seem to have been underestimated. It is also important to note that transgender men and gender-fluid individuals are affected, highlighting the importance of inclusive care and gender-neutral language.

POI and early menopause can lead to problematic menopausal symptoms and affect bone and cardiovascular health, sexual function and cognition (Faubion *et al.* 2015, Sochocka *et al.* 2023). The psychological impact is also considerable, including increased risks of mood disorders, such as depression and anxiety, as well as infertility-related distress, which further impair quality of life (Sterling & Nelson 2011, Faubion *et al.* 2015, Tian *et al.* 2024).

Hormone replacement therapy (HRT) is the cornerstone of management for both POI and early menopause. It is recommended until the average age of natural menopause, helping with symptom control and mitigating the long-term health risks associated with oestrogen deficiency (Sullivan *et al.* 2016).

This review aims to comprehensively outline the aetiology of POI and early menopause, describe the long-term health consequences and delineate key priorities for future research to improve diagnosis, management and patient outcomes.

## Aetiology of POI and early menopause

Although POI and early menopause are fairly common in the population, in many cases, the exact cause is not found. Studies usually group causes of POI into genetic, autoimmune, environmental, iatrogenic and idiopathic. The exact prevalence of each aetiology remains unknown, as most reported proportions derive from clinical settings rather than population-based studies. A study from a single centre compared the aetiologies in a historical cohort of women with POI with those in a contemporary cohort (Csehely *et al.* 2025*a*). Over time, an increase in iatrogenic and autoimmune cases was noticed, with a reduction in idiopathic POI, while the prevalence of genetic aetiology was unchanged.

With regard to early menopause, the aetiology is multifactorial and can often overlap with that of POI, with often no single cause found in many patients. Lifestyle factors, such as lower childhood socio-economic status, smoking and shorter time breastfeeding, can also be linked to higher risk of menopause before the age of 45 years (Peycheva *et al.* 2022).

We outline the most common aetiologies of POI and early menopause, along with key risk factors, in the subsections below.

### Genetic factors

A family history of premature menopause is a well-established risk factor for POI and early menopause, likely reflecting monogenic or oligogenic inheritance of susceptibility genes. Some of these are those involved in X-chromosome defects, the most common of which are Turner’s syndrome and *FMR1* premutation carrier status (Qin *et al.* 2015). *FMR1* premutation carriers display higher than normal levels of messenger RNA (Tassone *et al.* 2007); however, the mechanism by which this influences the phenotype, particularly the development of POI, remains unclear (Qin *et al.* 2015, Rosario & Anderson 2020).

Galactosaemia, a rare inherited metabolic disorder, is strongly associated with POI, with the majority of affected people developing ovarian insufficiency despite dietary galactose restriction. The mechanism is thought to involve toxicity from accumulated galactose-1-phosphate, which accelerates oocyte apoptosis (Thakur *et al.* 2018).

Recent advances in gene discovery have identified additional rare variants (Heddar *et al.* 2022), while polygenic risk scores derived from genome-wide association studies also contribute to heritability (Perry *et al.* 2013) but are not routinely tested in clinical practice, as they do not currently alter patient management.

### Autoimmunity

Autoimmunity is a significant contributor to POI and early menopause, but it is variably estimated. Autoimmune disease is thought to coexist in a large number of POI patients, although this does not always imply direct ovarian autoimmunity. The most common associations are with thyroid disease and adrenal insufficiency (Szeliga *et al.* 2021). As such, screening for adrenal and anti-thyroid antibodies can be recommended as part of the investigations for spontaneous POI (Panay *et al.* 2024). Routinely performing ovarian autoantibody assays is not advised, as they carry a high risk of false-positive results (Szeliga *et al.* 2021, Panay *et al.* 2024).

### Environmental and lifestyle factors

Several environmental and lifestyle factors contribute to the development of early menopause. Beyond non-specific industrial chemical exposure, there is growing evidence for, and awareness of, a possible role of endocrine-disrupting chemicals (EDCs) in reproductive health. EDCs may directly affect the female reproductive system and contribute to ovarian ageing. A narrative review of animal and human studies found that several environmental pollutants, especially tobacco, phthalates, bisphenol A and pesticides, are associated with adverse ovarian function, including increased follicular depletion, pre-antral follicle atresia and earlier menopause onset, although few studies directly addressed POI (Vabre *et al.* 2017). Given the ubiquity of EDC exposure, this may plausibly contribute to POI and early menopause, but further dedicated research is warranted. Cigarette smoking in particular is a common exposure, and it may represent a frequent and modifiable risk factor for ovarian ageing and possibly POI, although the causal contribution to POI is not yet established (Vabre *et al.* 2017).

There seems to be an association between nulliparity and early ovarian decline, although the exact causal relationship is still not clear. In a pooled analysis of over 50,000 patients, nulliparous women have been found to be twice as likely to develop premature menopause and 30% more likely to experience early menopause compared with a multiparous cohort. A younger age at menarche is also a strong risk factor for POI and early menopause (Mishra *et al.* 2017).

Although infections such as mumps, tuberculosis or varicella have been linked to POI, there is no evidence of a causal relationship (Panay *et al.* 2024).

### Iatrogenic causes

Medical treatments, such as chemotherapy, radiotherapy and pelvic surgery, are established causes of POI and early menopause. The risk of developing POI after chemotherapy depends on the drug class, cumulative dose and age at the time of treatment (Anderson *et al.* 2020). Alkylating agents in particular, such as cyclophosphamide, pose a significant risk. Pelvic radiotherapy and total body irradiation frequently result in POI, due to ovarian tissue being highly sensitive to ionising radiation (Meirow 2001).

Surgery causes POI or early menopause by physical removal of ovarian tissue. Inadvertent damage of ovarian tissue can also occur with ovarian cystectomy, particularly for endometriomas. Prophylactic salpingo-oophorectomy is often performed as a risk-reducing surgery for *BRCA1* or *BRCA2* carriers and immediately causes surgical menopause (Pillay & Manyonda 2022).

### Idiopathic causes

A very high proportion of cases, likely the majority, of POI and early menopause remain unexplained despite extensive workup (Mishra *et al.* 2024, Panay *et al.* 2024).

## Diagnosis and clinical presentation

### Diagnostic criteria

The diagnosis of POI is based on oligomenorrhoea or amenorrhoea (which may be primary, meaning failure to establish menstruation, or secondary) for more than 4 months or a history of surgical menopause, together with persistently elevated gonadotrophins, before the age of 40 years. Two measurements of follicle-stimulating hormone (FSH) are often required to account for the possible intermittent reactivation of ovarian function. Diagnostic criteria and, in particular, FSH thresholds vary slightly across the European Society of Human Reproduction and Embryology (ESHRE), British Menopause Society (BMS) and National Institute for Health and Care Excellence (NICE) guidelines:

- ESHRE recommends the diagnosis of POI to be based on a 4-month history or more of disordered menstrual cycles and an FSH level of > 25 IU/L. One measurement is sufficient; however, assessment can be repeated after four to six weeks if there is any uncertainty (Panay *et al.* 2024).
- The BMS consensus statement defines POI as oligomenorrhoea or amenorrhoea for more than 4 months with two measurements of FSH > 40 IU/L four to six weeks apart (Hamoda 2024).
- In the NICE menopause guideline, the FSH threshold is set at > 30 IU/L and two measurements need to be performed four to six weeks apart (National Institute for Health and Care Excellence (NICE) 2026).

On the other hand, the diagnosis of early menopause is often clinical and retrospective (Mishra *et al.* 2024). Early menopause occurs between the ages of 40 and 45 years and is also characterised by menstrual disturbance and elevated FSH, although biochemical markers are not strictly required for diagnosis.

### Clinical manifestations

Symptoms of POI and early menopause arise from the early lack of oestrogen secondary to premature loss of ovarian function. They have interpersonal variation and can fluctuate due to residual and intermittent ovarian activity (De Vos *et al.* 2010). They may also depend on the underlying cause, as the symptoms which patients who have undergone surgical menopause report tend to be more abrupt and severe (Panay *et al.* 2024).

Vasomotor symptoms are very common and include hot flushes, heat intolerance and night sweats. Many suffer from genitourinary syndrome of menopause (GSM), a term describing the constellation of vulvovaginal and lower urinary tract symptoms attributable to hypo-oestrogenism. This can manifest with vaginal dryness, dysuria and urinary frequency. Sexual dysfunction can involve dyspareunia and loss of libido. Fatigue and joint pain are also common (Panay *et al.* 2020, 2024).

Psychological symptoms are not to be dismissed, as they can greatly impact quality of life. These can include mood swings, depression, anxiety, irritability, low energy and poor concentration (Singer 2019).

### Differential diagnoses

POI and early menopause are diagnoses of exclusion, and other causes of amenorrhoea and hypo-oestrogenism must be ruled out beforehand. Pregnancy is the most common cause of amenorrhoea and will always need to be excluded prior to more invasive tests.

Functional hypothalamic amenorrhoea (FHA) is a diagnosis of exclusion. It commonly results from stress, extreme weight loss, excessive exercise or a combination of these (Gordon *et al.* 2017). Distinguishing POI from FHA can be particularly challenging, as both conditions may present with amenorrhoea and hypo-oestrogenic symptoms in young women. The hormonal profiles, however, are characteristically different, with low oestrogen in both, but low or normal gonadotrophins in FHA, compared with persistently elevated FSH in POI. FHA also reflects broader neuroendocrine suppression secondary to energy deficiency and/or psychological stress, which can extend beyond the reproductive axis. There is a reduction not only in LH and FSH, but also often in tri-iodothyronine (T3), with further metabolic changes involving kisspeptin, leptin and insulin-like growth factor-1 (IGF-1) (Gordon *et al.* 2017, Saadedine *et al.* 2023).

In addition, hyperprolactinaemia, secondary to pituitary adenoma or certain drugs, thyroid disease and chronic systemic disease, for example, can also cause menstrual disturbance with non-menopausal-range gonadotrophins. Polyendocrine metabolic ovarian syndrome (PMOS) is a well-known cause of oligo-ovulation or anovulation; however, FSH will be normal and the picture is of hyperandrogenism and not hypo-oestrogenism (Teede *et al.* 2026). Outflow tract disorders or structural causes may lead to amenorrhoea, but will have a normal hormonal profile. History and clinical examination, prior to bloodwork and imaging, are crucial in ruling out some of these conditions (Balen *et al.* 2024).

### Investigations

As mentioned above, elevated FSH measurements are needed to diagnose POI and early menopause, before the age of 40 years and between the ages of 40 and 45 years, respectively. In addition, past medical and family history is essential, as they often provide indication of the aetiology. Clinical features of conditions associated with POI, such as the pathognomonic stigmata of Turner’s syndrome, can aid diagnosis and complement biochemical and genetic investigations (Panay *et al.* 2020). For Turner’s syndrome specifically, these may include short stature, a webbed neck, low-set ears and skeletal abnormalities. Recognising these on clinical examination should raise suspicion of this condition and prompt karyotype testing (Turner *et al.* 2025). Due to the limited evidence specifically on early menopause, it is reasonable that the workup should be similar to that of POI (Mishra *et al.* 2024).

Oestradiol would generally be found to be low in patients with POI and early menopause, although this is not specific to these conditions (Panay *et al.* 2024). Anti-Müllerian hormone (AMH), secreted by the granulosa cells of antral follicles and a marker of ovarian reserve, is frequently undetectable, indicative of diminished ovarian follicular activity (Visser *et al.* 2012).

Imaging, in the form of ultrasound, is not routinely utilised for the diagnosis of POI or early menopause, as often follicles are still visible. However, it may be valuable in the differential diagnosis of other conditions causing menstrual disturbance (Panay *et al.* 2024).

Some additional investigations can be helpful to diagnose non-iatrogenic POI. Genetic investigations are recommended, especially if the patient presents in adolescence or young adulthood with spontaneous POI. Karyotype testing can identify X chromosome abnormalities or aneuploidies, such as Turner’s syndrome. In particular, *FMR1* gene premutation is a known genetic cause of POI (Qin *et al.* 2015). There is an implication for future offspring, as male infants born to women who carry this mutation are at risk of fragile X syndrome. If any genetic abnormality is detected, specialist advice, genetic counselling and family testing are suggested. Testing for Y-chromosome material can be appropriate if clinical suspicion arises (Panay *et al.* 2024).

Autoimmune screening may be considered to further investigate the aetiology of POI and early menopause. Recommended investigations are adrenocortical autoantibodies, thyroid peroxidase autoantibodies and thyroid function tests. Routine ovarian autoantibody testing is not advised. ESHRE also recommends against routine screening for diabetes or infection (Panay *et al.* 2024).

## Long-term health consequences

### Cardiovascular and metabolic disease risk

The effect of POI and early menopause on cardiovascular and metabolic health is significant, given the premature loss of the cardioprotective effects of oestrogen. The risk of cardiovascular events in women suffering from POI was seen to be 1.3-fold (pooled-adjusted hazard ratio (HR) = 1.35, 95% CI: 1.06–1.63, *I*^2^: 0%) higher than women undergoing menopause after the age of 50 in a systematic review and meta-analysis (Behboudi-Gandevani *et al.* 2023). This represents a critical clinical issue given that cardiovascular disease is the leading cause of mortality among postmenopausal women (Schipper & Louwers 2020, Stevenson *et al.* 2021, Nash *et al.* 2022). This risk appears particularly pronounced for surgical POI. A UK Biobank cohort study of over 144,000 postmenopausal women found that surgical premature menopause (bilateral oophorectomy before the age of 40 years) was associated with a hazard ratio of 1.87 (95% CI:1.36–2.58) for a composite cardiovascular outcome, compared with a more modest hazard ratio of 1.36 (95% CI: 1.19–1.56) for natural premature menopause, after adjustment for cardiovascular risk factors and hormone therapy use (Honigberg *et al.* 2019).

Stroke risk also appears to be increased. In a pooled analysis of individual patient data from 15 observational studies across five countries and regions, women with premature natural menopause had a higher risk of a composite of stroke or coronary artery disease before the age of 60 years (HR: 1.88, 95% CI: 1.62–2.20), with the association attenuating at older ages (Zhu *et al.* 2019).

In another recent systematic review and meta-analysis, women with POI or early menopause were found to have a higher risk of type 2 diabetes, hyperlipidaemia, coronary heart disease, stroke and total cardiovascular events (Liu *et al.* 2023). Again, the risk seems to be more pronounced in women who have surgical menopause as a result of bilateral oophorectomy, possibly due to the abruptness of hormone withdrawal and the complete loss of ovarian endocrine function (Atsma *et al.* 2006, Liu *et al.* 2023).

Due to these considerations, the recommendation is to perform HbA1c and lipid profile at baseline and blood pressure and weight measurement annually (Panay *et al.* 2024).

### Bone health and osteoporosis

One of the most important consequences of early oestrogen deficiency is the impact on bone health. Oestrogen is fundamental to maintaining the balance between bone resorption and formation, and its early loss can lead to an accelerated decline in bone mineral density and increased risk of fractures (Nash *et al.* 2022, Meczekalski *et al.* 2023). Low dietary calcium, vitamin D deficiency, smoking and a lack of weight-bearing exercise can further compromise bone strength (Panay *et al.* 2020).

A recent retrospective study of over 100 women diagnosed with POI or early menopause assessed bone mineral density and DEXA results at baseline, along with the time between the last menstrual period and diagnosis to assess any delays. At the time of diagnosis, 43.1% of patients were diagnosed with osteopenia and 10.3% with osteoporosis. The delay in diagnosis and bone mineral density were negatively correlated (Csehely *et al.* 2025*b*), demonstrating the importance of early recognition and management of POI and early menopause for maintaining bone health.

In women with POI and likely also early menopause, oestrogen replacement is recommended from diagnosis until the usual age of menopause to prevent bone loss and reduce fracture risk and is the preferred first-line management of low bone mass in this population (Meczekalski *et al.* 2023, Panay *et al.* 2024). While both HRT and the combined oral contraceptive pill (COCP) were better than no treatment in a 2-year open-label randomised trial, HRT increased lumbar spine bone mineral density more than the COCP at 2 years (Cartwright *et al.* 2016). HRT is often regarded as being more physiological than the COCP, as HRT preparations usually contain oestradiol, whereas COCPs contain the synthetic ethinyl oestradiol and higher progestin doses (Sullivan *et al.* 2016).

Bisphosphonates are generally not recommended as first-line therapy in women of reproductive age with POI, given the paucity of long-term safety data in this population. They should only be considered with osteoporosis specialist input, with particular caution in women wanting a pregnancy. Unlike HRT, they also do not address the broader, non-skeletal consequences of hypo-oestrogenism (Panay *et al.* 2024).

A bone density scan with dual-energy X-ray absorptiometry (DEXA) should be therefore performed at baseline and may be repeated every two to five years, depending on the initial bone mineral density and the use of oestrogen replacement therapy (Panay *et al.* 2020, 2024).

### Cognitive outcomes and risk of dementia

The premature loss of oestrogen, which is a feature of POI and early menopause, seems to be linked to an increased risk of cognitive decline and dementia, including Alzheimer’s disease (Vujović *et al.* 2023). Oestrogen exerts neuroprotective effects through various mechanisms, including the promotion of neuronal growth, survival and synaptic plasticity (Sochocka *et al.* 2023).

A recent meta-analysis of eleven studies showed that women with early menopause and POI (OR: 1.37, 95% CI: 1.22–1.54; *I*^2^: 93%) had a greater risk of developing any type of dementia compared with women undergoing menopause at the average age (OR: 1.18, 95% CI:1.15–1.21; *I*^2^: 0%); however, the evidence is still limited and further research is needed (Karamitrou *et al.* 2023).

### Psychological and sexual health

POI and early menopause carry profound psychological and sexual health consequences that extend well beyond the vasomotor and physical symptoms described above. POI has been found to be associated with a significantly higher risk of developing depression and anxiety in a recent systematic review and meta-analysis, and women with POI also report poorer overall health-related quality of life than women with normal ovarian function (Li *et al.* 2020, Tian *et al.* 2024). Additionally, diagnosis of early-onset menopause, especially at a young age, can carry substantial psychological distress, particularly grief over the possible premature loss of fertility (Sterling & Nelson 2011, Singer 2019).

Sexual function can also be affected and is influenced by both biological and psychosocial factors. A systematic review and meta-analysis found that women with POI have moderately reduced sexual function across all domains of the Female Sexual Function Index compared with controls, most markedly with respect to dyspareunia. Notably, this pattern persisted in studies with women using HRT, suggesting that psychological and psychosexual factors, in addition to hypo-oestrogenism, likely contribute (Van Zwol-Janssens *et al.* 2024). Vulvovaginal atrophy and poor lubrication are caused by oestrogen deficiency, and this often contributes to low libido and vice versa (Nappi *et al.* 2019).

## Management

### Hormonal therapy

Systemic hormonal therapy is the mainstay of treatment for POI and early menopause. It helps to alleviate the troublesome symptoms associated with menopause and prevents the long-term complications detailed above (Sullivan *et al.* 2016, Panay *et al.* 2020, 2024). Hormonal therapy should be started immediately at diagnosis and continued until the age of natural menopause, albeit individualised according to factors such as residual ovarian activity, contraceptive requirements, uterine status and route and dose of administration. It should also take into account specific clinical circumstances, for example a personal history of breast cancer or other contraindications to hormone therapy, which may necessitate a modified or non-hormonal approach. NICE recommends that patients should be promptly referred to experienced healthcare professionals, such as reproductive medicine or menopause specialists, so that both physical and psychosocial aspects of the condition are managed appropriately and that they are offered individualised treatment (NICE 2026). The most commonly prescribed treatments for young patients are combined oral contraceptives (COCs) and hormone replacement therapy (HRT). Both are effective at alleviating the primary symptoms of oestrogen deficiency, such as vasomotor symptoms and psychological symptoms. In addition, topical vaginal oestrogen is highly effective for treating urogenital symptoms and has an excellent safety profile owing to minimal systemic absorption (Sullivan *et al.* 2016).

COCs are commonly available and usually contain 30 μg ethinyl oestradiol with a progestogen. It is preferable for people with POI or early menopause to take COCs continuously, to minimise oestrogen-free breaks, and stop for a few days when spotting occurs to allow for a withdrawal bleed (‘flexible-extended use’) (Nash *et al.* 2020). One of the main benefits of COCs for POI is that they are more familiar to younger individuals and they might carry less social stigma. An additional benefit is that COCs provide reliable contraception.

Although COCs and HRT are both effective and widely used, growing evidence supports a preference for HRT, particularly transdermal 17β-oestradiol, as first-line therapy over the COCP, other than for women with a strong personal preference for it. Ethinyl oestradiol-containing COCPs are strongly associated with increased hepatic sex hormone-binding globulin (SHBG) synthesis, which in turn lowers free testosterone levels. As low libido is already a common and important concern in women with POI and early menopause, this SHBG-driven reduction in free androgens may exacerbate sexual dysfunction in some women, providing a further, specific rationale for favouring transdermal HRT, which has a comparatively neutral effect on SHBG and androgen levels (Shifren *et al.* 2007, Zimmerman *et al.* 2014).

Oestrogen replacement via HRT is administered continuously and in different forms, for example transdermal (patch, spray or gel) or oral. The starting dose of oestrogen is usually 2 mg for oral HRT and for transdermal HRT, a 50 μg patch or 1.5 mg for the gel (2 pumps). In people with a uterus, progestogen is required for endometrial protection, either oral, such as micronised progesterone, or local, such as a levonorgestrel-releasing intrauterine system (LNG-IUS; Mirena®, Bayer, Germany), which can also provide contraception. Combined HRT preparations offer simplified usage (Nash & Davies 2024).

Evidence suggests that HRT, particularly regimens using physiological 17β-oestradiol, might provide better cardiovascular protection compared with COCs. This further reinforces the preference for this type of HRT as described above. Treatment with HRT resulted in lower mean 24-h systolic and diastolic blood pressure compared with COCPs in a study performed on 34 women undergoing treatment for 12 months (Langrish *et al.* 2009). Physiological oestrogen also seemed to reduce LDL and increase HDL levels in a study performed comparing 10 girls with Turner’s syndrome with 20 healthy controls (Taboada *et al.* 2011).

HRT also seemed to perform better at increasing bone density in 2 years compared with COCs in a small randomised controlled trial (Cartwright *et al.* 2016). Unlike the use of HRT after the average age of menopause, HRT in POI and early menopause does not seem to increase breast cancer risk above that of the general population (Collaborative Group on Hormonal Factors in Breast Cancer 2019).

The Premature Ovarian Insufficiency Study of Effectiveness of hormonal therapy (POISE) study, a large multicentre randomised controlled trial, is aiming to investigate the optimal hormone therapy strategy for people suffering from POI by comparing COCs with HRT, looking at short- and long-term outcomes (https://www.poise.ac.uk/home.aspx).

### Lifestyle interventions and supportive care

Lifestyle measures are crucial to optimise health outcomes in patients with POI and early menopause. Regular weight-bearing exercise, such as weight lifting, is recommended to ensure good bone mass and counteract the rapid bone loss associated with hypo-oestrogenism (Panay *et al.* 2020, 2024).

A healthy, balanced diet rich in calcium and vitamin D is also vital for skeletal health and maintaining a normal BMI (Silva *et al.* 2021). More specifically, women with POI and early menopause should aim for a calcium-replete diet, with a total calcium intake of approximately 1 g/day from dietary and, where necessary, supplemental sources. A dietary calcium calculator can be used to assess adequacy of intake, with supplementation considered where dietary intake is insufficient, especially for vegetarian or vegan diets. Vitamin D status should also be assessed and optimised, given its central role in calcium absorption and bone health, with supplementation recommended where levels are inadequate (Panay *et al.* 2024).

Effective management of POI can require a multidisciplinary approach to integrate support for mental health. Peer support networks, such as the Daisy Network (https://daisynetwork.org/) in the UK, can also provide a platform for women to share their experiences and reduce the isolation often felt after diagnosis.

Although a healthy lifestyle has been shown to prevent chronic diseases and enhance quality of life in postmenopausal women, evidence pertaining specifically to women with POI and early menopause remains limited and targeted research is needed to identify the most effective interventions to mitigate risks (Yeganeh *et al.* 2025).

While non-hormonal medications and supplements are not recommended and should not be considered as first-line treatment, unless there are any contraindications to either COCs or HRT, a clear distinction should be drawn between hormonal therapy, which is required to address the long-term systemic consequences of hypo-oestrogenism, and non-hormonal treatment of individual symptoms, which has a role specifically when hormonal therapy is contraindicated, unsuitable or declined. These treatments may include herbal supplements and other drugs, such as selective serotonin reuptake inhibitors (SSRIs) or bisphosphonates (Panay *et al.* 2020, 2024, Mishra *et al.* 2024, NICE 2026).

### Fertility preservation and reproductive options

To date, no medical intervention has been demonstrated to restore ovarian function or fertility in patients with established POI or early menopause. All family-building options should be presented in a balanced manner to support the individual’s long-term well-being (Baker 2011). Given the hypothesised spontaneous conception rate of 5% in POI (Van Kasteren *et al.* 1999, Fraison *et al.* 2019), it is reasonable to prescribe women who are trying to conceive HRT instead of COCs, as it is not contraceptive.

Following diagnosis, oocyte retrieval for cryopreservation or IVF is not usually feasible, due to the ovarian response to stimulation often being reduced or even absent. This should, however, be considered on a case-by-case basis. Given that intermittent ovarian activity can occur in POI, successful IVF pregnancies using a woman’s own oocytes have been reported in selected women (Fraison *et al.* 2019). Women who wish to pursue this route should be counselled honestly about the low, but non-zero, chance of success. Oocyte or embryo donation is therefore the most reliable available option for women who want to carry their own pregnancy when treatment with autologous oocytes is unsuccessful or not pursued (Anderson *et al.* 2020, Panay *et al.* 2024).

Fertility preservation is effective in patients before iatrogenic insults, such as chemotherapy or pelvic radiotherapy, or pelvic surgery, such as bilateral oophorectomy, or before the overt onset of POI, such as in cases of early genetic screening. Depending on the age, pubertal status and pathology, oocyte, embryo or ovarian tissue cryopreservation can be performed (Anderson *et al.* 2020).

## Future directions in research

Research on POI and early menopause should be focused on unravelling the molecular landscape of these conditions, to enable earlier detection and ‘rescue’ of the remaining ovarian reserve. Modern genetic testing strategies have the potential to uncover new susceptibility loci and causative genes (Qin *et al.* 2015, Heddar *et al.* 2022). Some recent studies utilising RNA sequencing and machine learning have identified specific candidate biomarkers, such as those related to the PI3K–AKT pathway, oxidative phosphorylation and DNA damage repair (Yu *et al.* 2023, Wu *et al.* 2024). Future efforts should aim to integrate genetic, epigenetic and environmental data to understand the interactions leading to early ovarian failure.

Among the emerging therapies, stem cell therapy and targeted pharmacological interventions are interesting, but still in the experimental phase (Polonio *et al.* 2021, Moustaki *et al.* 2023). Johnson *et al.* have reviewed novel strategies and used mathematical modelling to evaluate how slowing loss of primordial follicles or boosting their number could extend ovarian function and delay menopause in people at risk of POI (Johnson *et al.* 2025).

With regard to fertility preservation, given their limited applicability in established ovarian insufficiency, emerging strategies such as *in vitro* maturation (IVM) and application of oogonial stem cells in ovarian tissue should be explored (Anderson *et al.* 2020, Telfer & Andersen 2021). The integration of fertility-sparing surgical approaches and less gonadotoxic chemotherapy regimens in oncology should also be researched and prioritised.

There is currently a lack of robust data specific to patients with POI and early menopause, despite growing awareness. There is no optimal hormone replacement regimen and the ongoing POISE trial represents a step towards this (https://www.poise.ac.uk/home.aspx). The development of effective non-hormonal interventions is equally important for women for whom hormonal therapy is contraindicated or declined.

Finally, the psychosocial burden of POI and early menopause is under-recognised and more qualitative studies are needed. Standardised, evidence-based psychological support pathways integrated into routine clinical care are needed to address the grief, confusion and anxiety that many people, especially adolescents and young adults, experience following diagnosis (Sterling & Nelson 2011).

## Conclusion

POI and early menopause carry consequences that extend beyond reproductive health, affecting cardiovascular, skeletal, cognitive and psychological well-being. They have a heterogeneous aetiology, which spans genetic, autoimmune, iatrogenic and environmental factors, with a substantial proportion of cases remaining idiopathic despite thorough investigations (Panay *et al.* 2020, 2024).

Early and accurate diagnosis is important, as delays can increase the long-term health burden, particularly with respect to bone density loss and cardiovascular risk. For this reason, hormonal therapy, which remains the gold standard management, should be started promptly at diagnosis and continued until the average age of natural menopause. Lifestyle measures and psychological support should be incorporated to optimise overall health and well-being.

It should be acknowledged that much of the evidence underpinning these consequences and their management derives from observational cohorts and small trials, with few adequately powered randomised controlled trials specific to POI and early menopause. Management recommendations are therefore frequently extrapolated from studies conducted in postmenopausal women or based on expert consensus, and this evidence base should be borne in mind when applying recommendations to individual patients.

Despite recent advances, there are still critical gaps in the understanding of the underlying mechanisms and optimal treatment strategies. Time and resource investment in high-quality, inclusive research is urgently needed to improve outcomes for people with POI and early menopause globally.

## Declaration of interest

The authors declare that there is no conflict of interest that could be perceived as prejudicing the impartiality of the work reported.

## Funding

This research did not receive any specific grant from any funding agency in the public, commercial or not-for-profit sector.

## Author contribution statement

LC drafted the review. MCD revised it critically and approved the final version. Both authors approved the submitted manuscript.
